# MODERATING ROLE OF NEIGHBORHOOD ENVIRONMENT IN ASSOCIATIONS BETWEEN HEARING LOSS AND COGNITION AMONG OLDER ADULTS

**DOI:** 10.1093/geroni/igae098.2953

**Published:** 2024-12-31

**Authors:** Ethan Siu Leung Cheung, Zhe Zhang

**Affiliations:** University of Utah, Salt Lake City, Utah, United States; Columbia University, New York, New York, United States

## Abstract

The relationships between hearing loss and late-life cognitive challenges have been documented. However, little is known about how the relationships may be moderated by the built and social neighborhood environments. This study examined the associations between hearing loss and cognitive challenges among community-dwelling older adults and whether neighborhood characteristics (physical disorder and low social cohesion) moderated the associations. Cross-sectional national data from Round 11 of the National Health and Aging Trends Study were adopted (N = 2,515). Multinomial logistic regressions were used to examine associations among variables and interactive analyses were conducted to examine moderating effects. Results indicated significant relationships between the experience of hearing loss and possible dementia and between severe or profound hearing loss and probable dementia. Interactive models suggested that residing in neighborhoods with physical disorder and low social cohesion were negatively associated with possible dementia among older adults with moderate and severe or profound hearing loss, respectively, compared to those without hearing loss. Findings underscore the necessity of environmental and social interventions to enhance cognitive health among older adults with varying degrees of hearing challenges.

